# Will introduction of ARNI reduce the need of device therapy in heart failure with reduced ejection fraction?

**DOI:** 10.1186/s43044-021-00152-x

**Published:** 2021-03-16

**Authors:** Navjyot Kaur, CR Pruthvi, Manojkumar Rohit

**Affiliations:** Cardiology Unit II, Department of Cardiology, Level-3, Faculty Offices, Advanced Cardiac Center, Post Graduate Institute of Medical Education & Research, Sector-12, Chandigarh, 160012 India

## Background

The last two decades saw major therapeutic advancements in the field of heart failure with reduced ejection fraction (HFrEF). While implantable cardioverter-defibrillator (ICD) and cardiac resynchronization therapy (CRT) became part of HFrEF therapy in the first decade of twentieth century, the second decade marked the introduction of angiotensin receptor neprilysin inhibitor (ARNI) in optimal medical therapy (OMT) for HFrEF. ICD prevents sudden cardiac deaths (SCDs), and CRT improves left ventricular ejection fraction (LVEF), reverses cardiac remodeling, and decreases heart failure (HF)-related hospitalizations and mortality. While ICD prevents 50–60% SCDs but not all, the CRT has shown its consistent efficacy only in selected subset of HFrEF patients and has a non-responder rate of 30% [1]. The novel pharmacological therapy ARNI reduced the HF-related mortality and hospitalization when compared with well-established angiotensin-converting enzyme inhibitor (ACEi) [2]. Though ARNI reduced mortality both due to HF progression and SCD, it was associated with a higher incidence of symptomatic hypotension. In this opinion paper, we discuss the two very recent contemporary therapies (device therapy and ARNI) for HFrEF, the gap in evidence, and the projected role of each therapy in coming years.

## Important trials

### ICD

The role of ICD in primary prevention was established by Multicenter Automatic Defibrillator Implantation II (MADIT II) and Sudden Cardiac Death–Heart Failure trial (SCD-HeFT) trials [3]. While MADIT II included only ischemic patients with LVEF ≤ 35%, the SCD-HeFT trial included patients with both ischemic and non-ischemic etiology. However, Prophylactic Defibrillator Implantation in Patients with Non-ischemic Dilated Cardiomyopathy (DEFINITE) and Danish Study to Assess the Efficacy of ICDs in Patients with Non-Ischemic Systolic Heart Failure on Mortality (DANISH) trials failed to show a significant reduction in total mortality in non-ischemic cardiomyopathy (NICM), though the SCDs were reduced in both trials [1]. In contrast to SCD-HeFT where the use of beta blockers was less than 70%, more than 85% of patients in DEFINITE and DANISH trials were on beta blockers [4]. A recent meta-analysis of 06 randomized controlled trials (RCTs) involving 2970 patients with NICM revealed 23% risk reduction in all-cause mortality with ICD [5], and hence, the present-day guidelines recommend the use of ICDs for primary prevention in HFrEF (New York Heart Classification (NYHA)-II/III) irrespective of the etiology. However, ICD is not advisable in patients with NYHA class IV symptoms or whose expected meaningful survival is less than 1 year.

### CRT

Electromechanical dyssynchrony leads to non-synchronized left ventricular contraction, adverse cardiac remodeling, and increased myocardial energy expenditure. While Multisite stimulation in Cardiomyopathy (MUSTIC-SR), Multicenter In Sync Randomized Clinical Evaluation (MIRACLE), and CONTAK CD trials showed a significant improvement in NYHA functional class and peak oxygen consumption (pVO2) in selected patients of HFrEF (NYHA classes III and IV) with CRT, the subsequent two trials, Cardiac Resynchronization in Heart Failure Trial (CARE HF) and Comparison of Medical Therapy, Pacing, and Defibrillation in Heart Failure (CHAMPION) trials, provided evidence for a reduction in mortality and HF hospitalizations in same functional class of patients with HFrEF [6]. CRT in moderate to severely symptomatic HFrEF patients reduces the all-cause mortality by 28% and HF hospitalizations by 37% [7]. The role of CRT in mildly symptomatic patients (NYHA II) was established by Resynchronization Reverse Remodelling in Systolic Left Ventricular Dysfunction trial (REVERSE), MADIT-CRT, and Resynchronization/Defibrillation for Ambulatory Heart Failure Trial [RAFT] trials [6]. Based on available evidence, the present guidelines on HFrEF give the highest level recommendation for CRT in patients with LVEF ≤ 35% who have left bundle branch block (LBBB) and wide QRS ≥ 150 ms and remain symptomatic (NYHA class II, III, ambulatory IV) despite receiving OMT [6]. It is noteworthy that all the above mentioned RCTs were conducted between 2000 and 2010, when ARNI was not part of OMT. Moreover, the CRT has shown maximum and most consistent benefit only in a subset of patients with HFrEF and has a non-response rate of 30% even in this highly selected subgroup. Further, the CRT failed to show a consistent positive impact in HFrEF patients with non-LBBB and narrow QRS.

### ARNI

The pathophysiology of HFrEF is marked by up-gradation of the renin-angiotensin-aldosterone system and by down-gradation of cardio-protective molecules like natriuretic peptides. ARNI, the combination of angiotensin receptor blocker (ARB) and neprilysin inhibitor (sacubitril/valsartan), proved superior to well-established angiotensin-converting enzyme inhibitor (ACEi) and showed a 20% relative risk reduction in the primary endpoint of cardiovascular (CV) death and HF hospitalization and 16% reduction in all-cause mortality in the landmark PARADIGM-HF trial [2]. Both SCDs and deaths due to HF progression were reduced equally. Soon after, the American and European guidelines [8, 9] included ARNI into the pharmacological management of symptomatic HFrEF patients (NYHA II-IV) on an outpatient basis. PIONEER-HF trial demonstrated the safety of introduction of ARNI in patients with acute decompensated heart failure (after stabilization) [10]. The most significant side effect reported with ARNI has been symptomatic hypotension which may hamper the up-titration of the drug to target dosages and lead to high rates of non-compliance and non-adherence in real-world scenarios. There are concerns about decreased clearance and deposition of beta amyloid peptides in the brain due to neprilysin inhibition with long-term use of ARNI. Table 1 summarizes the major RCTs done on ARNI in the last decade.

**Table 1:** Summary of major randomized controlled trials on angiotensin receptor neprilysin inhibitor

Trial	Intervention	Comparator	Inclusion criteria	Primary endpoints	Outcomes
PARADIFM-HF (2014) (*n* = 8399) F/up 27 months	Sacubitril/vvalsartan	Enalapril	-LVRF ≤ 35 -NYHA classes II–IV -Elevated NP	Composite of CVD or HF hospitalization	Primary endpoint HR = 0.80 (0.73–0.87) *P* = 0.0000002 CVD HR = 0.80 (0.71–0.89) *P* = 0.00004 All-cause mortality HR = 0.84 (0.76–0.93) *P* < 0.0001 Symptomatic hypotension (14% vs 9.2%; *P* < 0.001)
TITRATION (2016) (*n* = 498) F/up 12 weeks	Condensed regimen (full dose of ARNI by 3 weeks)	Comparator regimen (full dose of ARNI by 6 weeks)	LVEF ≤ 35% NYHA classes II–IV	Adverse events (hypotension, renal dysfunction, hyperkalemia, angioedema)	No significant difference
PIONEER-HF (2019) (*n* = 881) F/up 8 weeks	Sacubitril/valsartan	Enalapril	LVEF ≤ 40% Elevated NP Hospitalized for ADHF	Time-averaged change of NT-proBNP	Primary endpoint HR 0.71 (0.63–0.81), *P* < 0.05 HF rehospitalization 8.0 % vs. 13.8%, *P* < 0.05
PARAMOUNT (2012) (*n* = 301) F/up 12 weeks	Sacubitril/valsartan	Valsartan	LVEF ≥45% NYHA classes II–III Elevated NP	Change in NT-pro BNP from baseline	Significant change in favor of ARNI: Ratio 0.77 (0.64–0.92)
PARAGON-HF (2019) (*n* = 4822) F/up 35 months	Sacubitril/valsartan	Valsartan	LVEF ≥ 45% NYHA classes II–III Elevated NP	Composite of HF hospitalizations and CVDs	Non-significant Relative risk 0.87 (0.75–1.01)
EVALUATE-HF (2019) (*n* = 464) F/up 12 weeks	Sacubitril/valsartan	Enalapril	LVEF ≤40% NYHA classes I–III History of hypertension	Aortic characteristic impedance (Zc)	Non-significant difference in two groups
PRIME (2019) (*n* = 118) F/up 12 months	Sacubitril/valsartan	Valsartan	LVEF 25–50% NYHA classes I–III Chronic functional MR (EROA > 0.1 cm^2^ despite optimal medical therapy)	Change in EROA	Significant decrease in EROA (∇ 0.04cm^2^), regurgitant volume (∇ 7.3 ml), LVEDVI (∇ 7.01 ml)
PROVE-HF (2019) (*n* = 794) F/up 12 months	Sacubitril/valsartan	ACEi/ARB	LVEF ≤ 40% NYHA II–IV	Correlation between change in NT-pro BNP and remodeling (LVEF, LVEDVi, LVESVi, LAVi, E/E/e' at 12 months	Significant correlations observed between the change in NT-proBNP concentration and all cardiac remodeling parameters

*ACEi* Angiotensin-converting enzyme inhibitor, *ADHF* Acute decompensated heart failure, *ARB* Angiotensin receptor blocker, A*RNI* Angiotensin receptor neprolysin inhibitor, *CV* Cardiovascular, *CVD* Cardiovascular death, *E/e'* Ratio of early mitral diastolic filling velocity/early diastolic mitral annular velocity, *EROA* Effective regurgitant orifice area, *EVALUATE-HF* Effect of Sacubitril-Valsartan Versus Enalapril on Aortic Stiffness in Patients with Heart Failure and Reduced Ejection Fraction, *F/up* Follow-up, *HF* Heart failure, *HR* Hazard ratio, *LAVi* Left atrial volume index, *LVEDVi* LV end-diastolic volume index, *LVEF* Left ventricular ejection fraction, *LVESVi* LV end-systolic volume index, *MR* Mitral regurgitation, *NP* Natriuretic peptide, *NT-proBNP* N-terminal-pro B-type NP, *NYHA* New York Heart Association, *PARADIGM-HF* Prospective comparison of Angiotensin Receptor-Neprilysin Inhibitor (ARNI) with ACEI to Determine Impact on Global Mortality and morbidity in Heart Failure, *PARAGON-HF* Prospective Comparison of ARNI with ARB Global Outcomes in HF With Preserved Ejection Fraction, *PARAMOUNT* Prospective comparison of ARNI with ARB on Management Of heart failure with preserved ejection fraction, *PIONEER-HF* Comparison of Sacubitril-Valsartan versus Enalapril on Effect on NT-proBNP in Patients Stabilized from an Acute Heart Failure Episode, *PRIME* Pharmacological Reduction of Functional, Ischemic Mitral Regurgitation, *PROVE-HF* Prospective Study of Biomarkers, Symptom Improvement and Ventricular Remodeling During Entresto Therapy for Heart Failure, *RFT* Renal function test, *∇* Change

## CRT and ARNI: cardiac remodeling

CRT is considered one of the most powerful cardiac remodeling agents, second only to beta blockers [4]. It reverses the adverse cardiac remodeling, decreases LV and left atrial dimensions, and improves LVEF and functional mitral regurgitation, and the same has been co-related with positive clinical outcomes in MADIT-CRT and REVERSE trials [6]. The effect of ARNI on cardiac remodeling has been recently illustrated in PROVE HF trial [11] wherein ARNI over a period of 12 months improved LVEF by 9.4% and reduced LV end-diastolic volume index, LV end-systolic volume index, and left atrial volume index. A recent Italian observational SAVE ICD study by Federico Guerra revealed that after 6 months of treatment with ARNI, 25% of patients with ICD for primary prevention had LVEF ≥ 35% [12].

We cannot compare the absolute effect on cardiac remodeling between two modalities because of the heterogeneities of populations studied. Also, the CRT is offered over and above the OMT and reduction in LV volumes and improvement in LVEF can be at the best considered add-on, rather than an absolute effect. On the other hand, ARNI has proven to be effective when compared with another well-established RAAS blocking agent [2].

## CRT and ARNI: do they reduce the risk of SCD and ventricular arrhythmias (VA)?

The CHAMPION trial failed to show significant mortality benefit of CRT-D over CRT-P [1]. In a more recent CeRtiTude cohort study, the rate of SCD was no different in two groups of patients with CRT-P and CRT-D at 2-year follow-up, despite the fact that the patients in the former group were sicker and older and had multiple comorbidities [13]. A review by Barra et al. in 2019 revealed that the risk of SCD in CRT patients has reduced more than fourfold over the last 20 years; the rate of decline in mortality including SCD has been more in the CRT-P group and it related closely to increased LVEF, increased use of beta blockers, decreased QRS duration, and decreased use of antiarrythmic drugs [14]. The PARADIGM trial showed an impressive reduction of 22% in the rate of SCDs [2]. de Diego et al. [15] and Martens et al. [16] revealed a significant reduction in sustained and non-sustained VAs and appropriate ICD shocks in the ARNI group and the latter also documented better pacing parameters of ICDs in patients on ARNI [17]. Sacubitril/valsartan reduces the arrhythmia burden primarily by improvement in cardiac remodeling, though the smaller studies have also illustrated a decrease in QRS duration, QTc interval, and mechanical dispersion as assessed by LV global longitudinal strain imaging [﻿﻿﻿^18^].

## The GAP in evidence

CRT and ICD have shown to reduce mortality and morbidity in a selected subgroup of HFrEF patients who remain symptomatic while on OMT. ARNI improves clinical outcomes as compared to the well-established ACEi. Whether these devices would have significant positive clinical outcomes when used along with ARNI is still not known. In the case of ICDs, the meaningful reduction of mortality when used for primary prevention can only occur if the baseline risk of SCD is > 35% or the rate of SCD ≥ 1.2% per year [1]. With the increased use of beta blockers, the absolute risk of SCDs reduces and the additional ICD may have insignificant benefit [1, 4]. Similarly, with the addition of ARNI, the absolute CV mortality may reduce with no added benefit of any device therapy. Since ARNI is associated with a significantly increased risk of hypotension, compliance with the drug and achievement of target dosage may be an issue in real-world scenario especially in the Asian population. Likewise, the clinical benefit achieved with suboptimal dosages needs to be studied in prospective registries.

## ARNI and devices: two complementary therapies in HFrEF

**Fig. 1:**
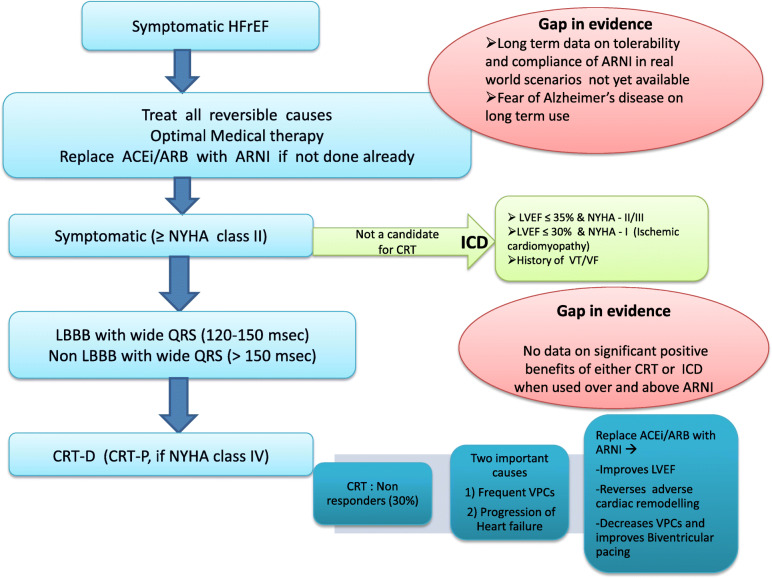
Proposed algorithm for management of heart failure with reduced ejection fraction

ARNI can be used in all symptomatic patients of HFrEF while CRT has shown maximum benefit in patients with LBBB and wide QRS. It causes time-dependent improvement in LVEF [11, 12], may reduce the need of ICD for primary prevention [12], and has shown to improve the biventricular pacing by reducing ventricular ectopy and thus can improve the percentage of responders in the CRT group. ARNI may also reduce the appropriate ICD therapy in patients with HFrEF by reducing VAs. However, till the time a robust evidence draws an entirely different conclusion, we should follow the present guidelines on the appropriate use of ICD, CRT, and ARNI in patients with HFrEF, though we may use ARNI more often as part of OMT with an aim to achieve the best possible benefit. Figure 1 illustrates the respective role of various therapies in the management of HFrEF.

## Conclusion

ARNI and device therapy for HFrEF are two complementary therapies. While ARNI and CRT improve LVEF, reduce LV volumes, and produce significant improvement in cardiovascular mortality and HF hospitalizations, the ICDs reduce the additional risk of SCD in selected patients. Given the evidence, the ARNI shall be the future foundation of OMT for HFrEF, with CRT/ICD reserved for special situations where patients remain symptomatic while on OMT. The role of CRT/ICD in the era of ARNI needs to be re-established since the definition of OMT stands changed with much better and potent pharmacological therapy.

## Data Availability

Not applicable for the above-said reasons

## Abbreviations

ACEi: Angiotensin-converting enzyme inhibitor
ARB: Angiotensin receptor blocker
ARNI: Angiotensin receptor neprilysin inhibitor
CRT: Cardiac resynchronization therapy
CV: Cardiovascular
HF: Heart failure
HFrEF: Heart failure with reduced ejection fraction
ICD: Implantable cardioverter-defibrillator
LBBB: Left bundle branch block
NICM: Non-ischemic dilated cardiomyopathy
NYHA: New York Heart Classification
OMT: Optimal medical therapy
pVO2: Peak oxygen consumption
RCTs: Randomized controlled trials
SCDs: Sudden cardiac deaths
